# Giant Lipoma of the Thoracic Wall in a Two-Year-Old Girl: Imaging Features Supporting a Benign Diagnosis

**DOI:** 10.7759/cureus.118987

**Published:** 2026-11-02

**Authors:** Gabriel Da Silva Eli, Marcelo Rizon, Ana A Bartolamei Ramos

**Affiliations:** 1 Diagnostic Radiology, Hospital de Clínicas Universidade Federal do Paraná, Curitiba, BRA; 2 Surgery, Hospital do Rocio, Curitiba, BRA; 3 Radiology, Universidade Federal do Paraná, Curitiba, BRA

**Keywords:** chest wall, giant lipoma, lipoblastoma, liposarcoma, magnetic resonance imaging, pediatric soft-tissue tumor

## Abstract

Lipomas are the most common mesenchymal neoplasms of soft tissue, but giant lipomas, defined as lesions exceeding 10 cm in any dimension or weighing more than 1 kg, are rare in young children, in whom lipoblastoma is the main adipocytic differential diagnosis and liposarcoma must be excluded. We report the case of a two-year-old girl with a left dorsal thoracic wall mass that had grown progressively over 18 months. A biopsy performed elsewhere one year earlier had shown mature adipose tissue without atypia. Radiography showed a large soft-tissue density mass with rib deformity, a sixth-rib fracture, and superior displacement of the scapula. Ultrasound demonstrated a solid, hypovascular mass with echogenicity similar to subcutaneous fat. Magnetic resonance imaging showed a 12.0 × 10.2 × 9.1 cm mass that followed fat signal, with signal loss on fat-suppressed sequences, no restricted diffusion, and no significant enhancement, extending through an intercostal space into the extrapleural compartment. At surgery, the tumor adhered to the chest wall muscles and ribs and extended into the sixth intercostal space, deforming the adjacent ribs and displacing the pleura and lung without invading them. It was dissected circumferentially and resected without complications. The specimen measured 13.2 × 10.1 × 10.0 cm and weighed 456 g, and histopathology showed a lipoma with a focus of ischemic necrosis and calcification. Because immunohistochemical and molecular studies were not performed, lipoma remains a presumptive diagnosis, and a mature lipoblastoma cannot be completely excluded. There was no clinical or imaging recurrence after one year of follow-up. This case illustrates imaging features that support a benign adipocytic tumor in early childhood and the limits of imaging, which cannot replace histological confirmation.

## Introduction

Lipomas are benign tumors composed of mature adipocytes and are the most common soft-tissue neoplasms [1]. They typically present as painless, slow-growing, soft, and mobile subcutaneous masses, most often on the trunk and proximal extremities, and are usually diagnosed in adults [1]. Most are superficial and smaller than 5 cm; lesions exceeding 10 cm in any dimension or weighing more than 1 kg are termed giant lipomas [2,3].

Adipocytic tumors are uncommon in early childhood, when the spectrum of soft-tissue masses differs substantially from that of adults [4]. In children younger than three years, lipoblastoma, a benign tumor of embryonal fat that may be clinically and radiologically indistinguishable from lipoma, is the leading consideration [5,6]. In any large fatty mass, the main concern remains the exclusion of well-differentiated liposarcoma. Magnetic resonance imaging (MRI) features such as size greater than 10 cm, thick septa, and nodular non-adipose components help identify lesions that warrant further investigation, but these criteria were derived mainly from adult series and do not allow a definitive distinction between benign and malignant adipocytic tumors [7].

Giant lipomas can cause compression of adjacent nerves, restricted joint motion, and cosmetic and functional impairment, and their size alone raises concern for malignancy [8]. Although malignant adipocytic tumors are exceptional in young children [9], a large or deep fatty mass in this age group requires careful evaluation before treatment. Reports of giant fatty tumors in children remain scarce and involve diverse sites, including the thoracoabdominal wall [3], the upper extremity of an adolescent [10], and the thoracic cavity, where a giant lipoblastoma occupying almost the entire left hemithorax was recently described in a one-year-old boy [11].

Palpable chest wall masses are a frequent reason for imaging in children, and most correspond to normal anatomic variants or benign lesions [12]. Imaging helps decide which children need cross-sectional evaluation or biopsy, but it cannot replace histological confirmation when a mass is large, deep, or growing [7,12].

We report a giant lipoma of the thoracic wall in a two-year-old girl that extended through an intercostal space into the extrapleural compartment and was associated with rib deformity and a rib fracture. This combination of very young age, size greater than 10 cm, and transmural chest wall extension with skeletal changes is unusual. We describe the imaging features that supported a benign diagnosis before resection, correlate them with the operative and pathological findings, and discuss the limitations of imaging in distinguishing lipoma from lipoblastoma and liposarcoma in early childhood.

## Case presentation

A previously healthy two-year-old girl was referred to the pediatric surgery department of the Hospital de Clínicas, Federal University of Paraná (Curitiba, Brazil), for a left dorsal mass that had grown progressively over the preceding 18 months. She had no pain, dyspnea, or other systemic symptoms. The mass had first been noticed by her mother at six months of age as a small palpable nodule. There was no history of trauma and no family history of multiple lipomas or other neoplasms. On physical examination, the mass measured at least 9 to 10 cm on palpation and was firm, slightly elastic, and fixed to the chest wall, with normal overlying skin. The scapula was displaced superiorly, and there were no other skeletal abnormalities. Breath sounds were reduced over the accessible left lung fields and normal on the right side. At approximately one year of age, a core needle biopsy had been performed at another facility in her hometown, when the mass was smaller, although its size at that time was not documented. It had shown mature adipose tissue without atypia. Because the biopsy was benign, no further follow-up was considered necessary at that time, and she was referred to our service months later because the mass continued to grow. Referral was further delayed because she lived in a town in the interior of the state, far from the capital, with limited access to pediatric surgical care, and because of the high demand for pediatric surgery in the Brazilian public health system (Sistema Único de Saúde). At admission, vital signs were within the normal range for age, and routine preoperative laboratory tests were unremarkable (Table 1).

**Table 1:** Vital signs and laboratory test results at admission. Reference ranges are age-specific values for a two-year-old child. SpO_2_: peripheral oxygen saturation; MCH: mean corpuscular hemoglobin; MCHC: mean corpuscular hemoglobin concentration; RDW: red cell distribution width; WBC: white blood cell; eGFR: estimated glomerular filtration rate; PT: prothrombin time; INR: international normalized ratio; aPTT: activated partial thromboplastin time

Parameter	Result	Reference range
Heart rate	110 beats/minute	98–140 beats/minute
Respiratory rate	22 breaths/minute	22–37 breaths/minute
Systolic blood pressure	90 mmHg	86–106 mmHg
Diastolic blood pressure	56 mmHg	42–63 mmHg
Temperature	36.5 °C	36.0–37.5 °C
SpO2 (room air)	>95%	≥95%
Capillary refill time	<2 seconds	≤2 seconds
Hemoglobin	12.1 g/dL	10.5–13.5 g/dL
Hematocrit	36%	33–39%
MCH	26 pg	23–31 pg
MCHC	34 g/dL	30–36 g/dL
RDW	12.5%	11.5–14.5%
WBC count	6,000/µL	5,500–15,500/µL
Neutrophils	1,500/µL	1,500–8,500/µL
Lymphocytes	3,000/µL	3,000–9,500/µL
Platelet count	180,000/µL	150,000–450,000/µL
Creatinine	0.3 mg/dL	0.2–0.4 mg/dL
Urea	12 mg/dL	11–39 mg/dL
eGFR	>90 mL/minute/1.73 m²	≥90 mL/minute/1.73 m²
Sodium	138 mEq/L	135–145 mEq/L
Potassium	4.0 mEq/L	3.5–5.0 mEq/L
Total calcium	9.0 mg/dL	8.8–10.8 mg/dL
Phosphorus	4.5 mg/dL	3.8–6.5 mg/dL
PT	11 seconds	10–14 seconds
INR	1.1	0.8–1.2
aPTT	30 seconds	25–35 seconds
Fibrinogen	300 mg/dL	200–400 mg/dL

Chest radiography showed a large soft-tissue density mass in the left thoracic wall, measuring 11.0 × 9.8 cm on the posteroanterior view, with widening of an intercostal space, deformity of the adjacent ribs, a slightly displaced fracture of the sixth rib with signs of ongoing consolidation, and superior displacement of the scapula (Figure 1).

**Figure 1:**
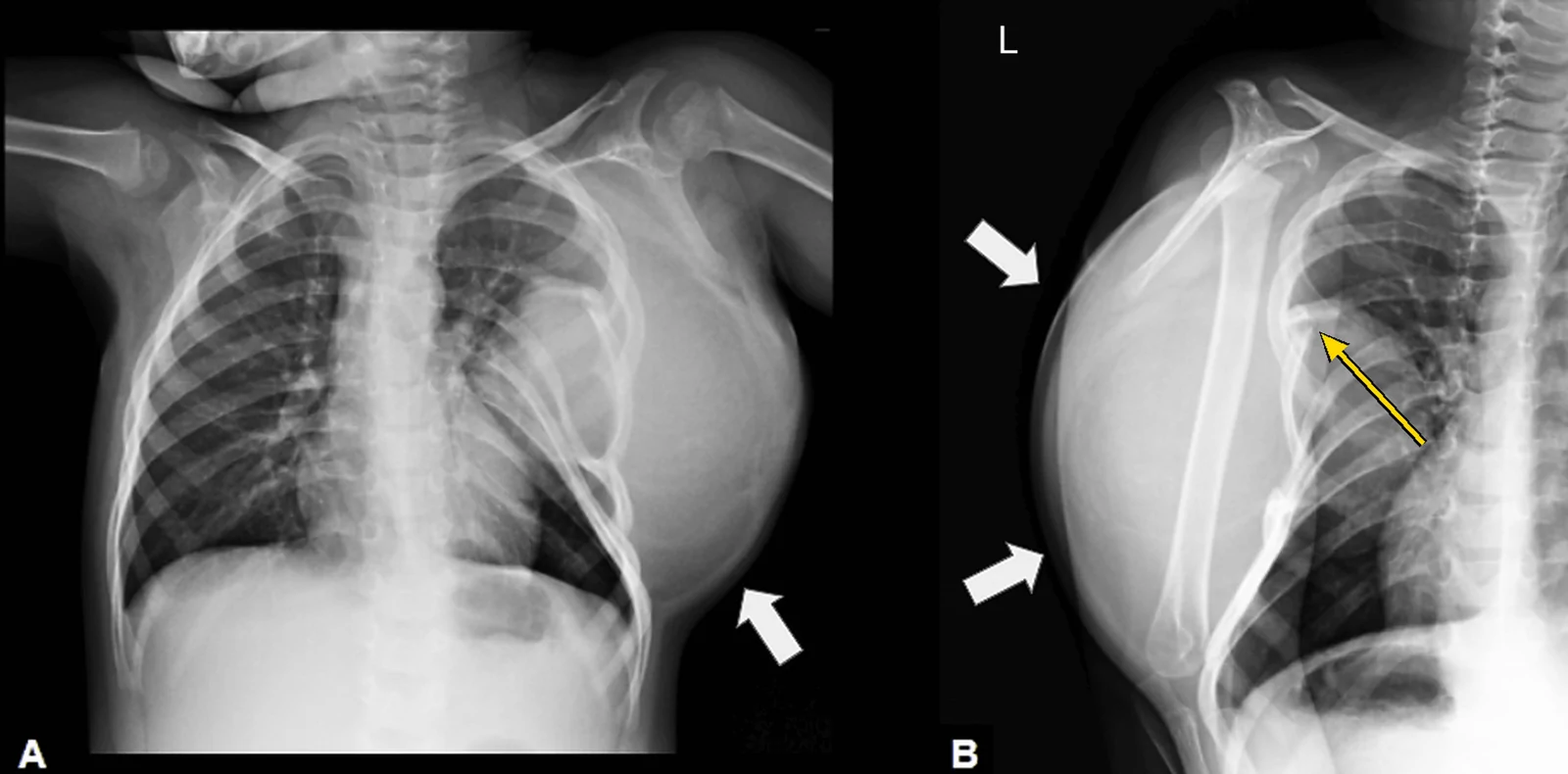
Chest radiography. Posteroanterior (A) and scapular profile (B) views show a large soft-tissue density mass in the left thoracic wall (white arrows), measuring 11.0 × 9.8 cm on the posteroanterior view. The yellow arrow in B indicates the slightly displaced fracture of the sixth rib with signs of ongoing consolidation. Note the deformity of the adjacent ribs and the superior displacement of the scapula. L: left side marker

Targeted ultrasound demonstrated a solid, slightly heterogeneous mass with echogenicity similar to subcutaneous fat and hypovascularity on color Doppler. The lesion exceeded the field of view, and only a partial measurement of 9.3 cm was possible (Figure 2).

**Figure 2:**
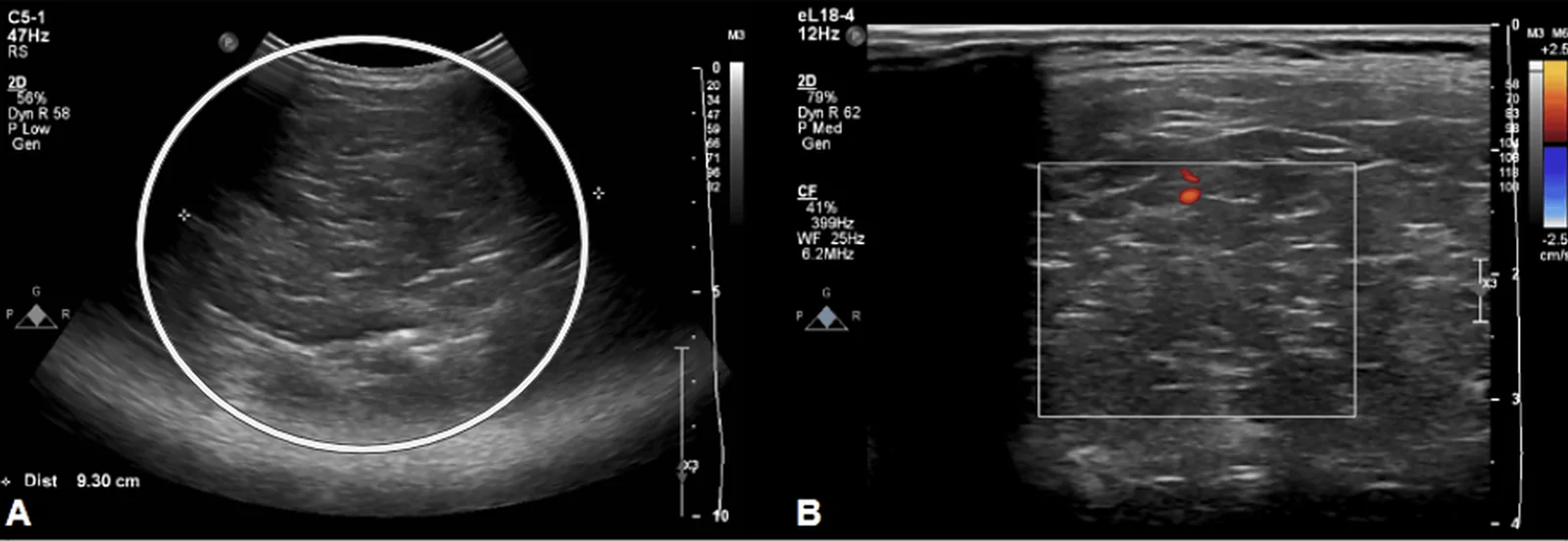
Ultrasound findings. Ultrasound of the left thoracic wall focuses to characterized the dorsal lesion, demonstrates a solid, slightly heterogeneous mass with an echogenicity predominantly similar to that of subcutaneous tissue (A, white circle) and hypovascularity on color Doppler (B), not fully measurable by this method.

Chest magnetic resonance imaging (MRI) showed a mass of approximately 12.0 × 10.2 × 9.1 cm that followed the signal of subcutaneous fat on all available sequences. It was predominantly hyperintense on T2-weighted images, with rare thin septa, showed homogeneous signal loss on short tau inversion recovery and fat-saturated T1-weighted images, had no restricted diffusion, and showed no significant enhancement on post-contrast fat-saturated T1-weighted images. The lesion extended through the sixth intercostal space into the extrapleural compartment of the left hemithorax, where its intrathoracic component measured approximately 8.0 cm in its largest dimension, displacing the pleura and lung without signs of pleural invasion, with remodeling of the adjacent ribs and passive atelectasis of the adjacent lung (Figure 3).

**Figure 3:**
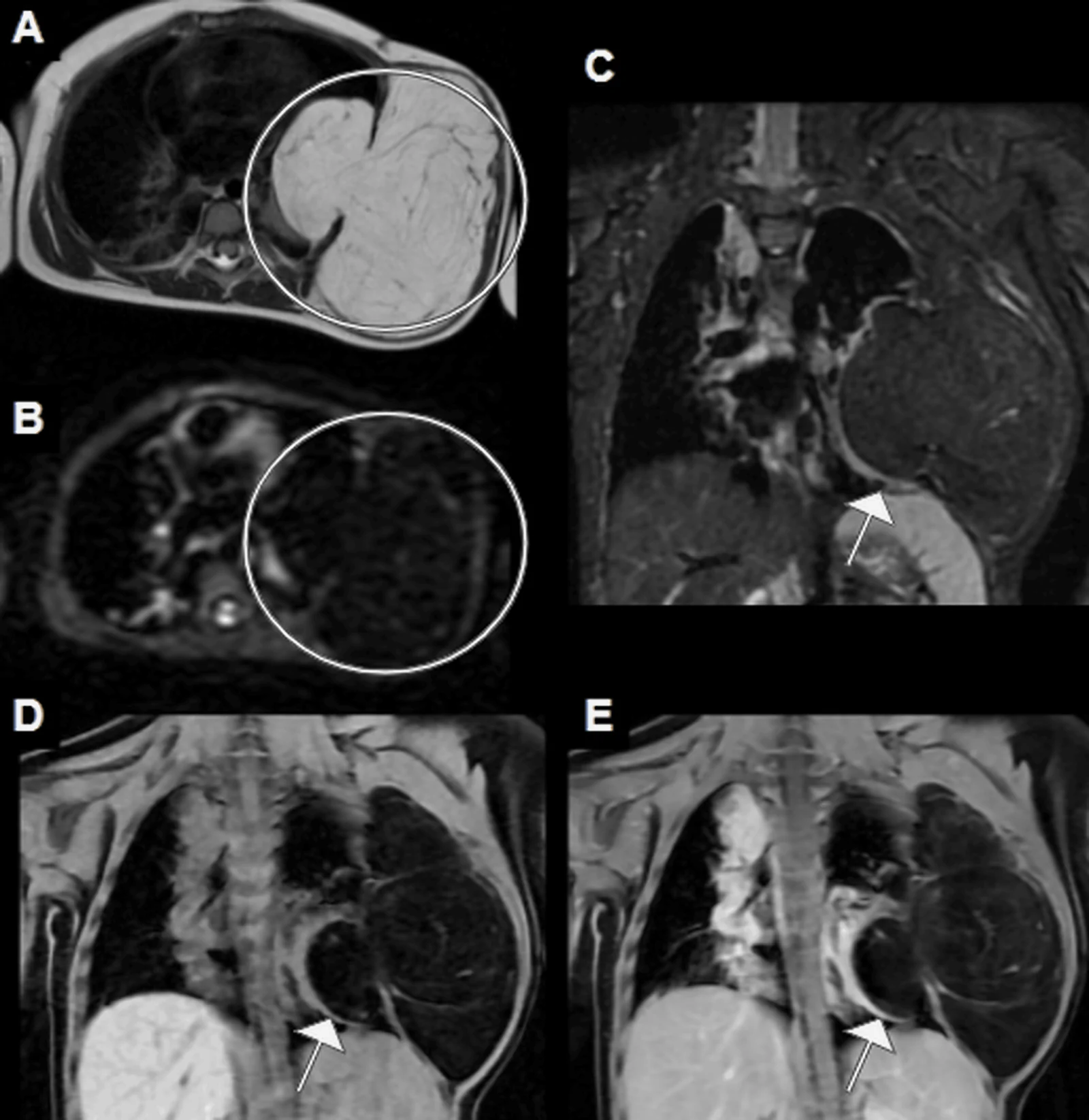
Magnetic resonance imaging of the chest. A large, predominantly fatty mass (white circle) with rare thin septa, hyperintense on the axial T2-weighted image (A), without restricted diffusion on the diffusion-weighted image (B), with homogeneous signal loss on the coronal short tau inversion recovery (C) and fat-saturated T1-weighted (D) images, and without significant enhancement on the post-contrast fat-saturated T1-weighted image (E). The mass measures approximately 12.0 × 10.2 × 9.1 cm and extends through the sixth intercostal space into the extrapleural compartment of the left hemithorax, where its intrathoracic component (thin white arrows) measures approximately 8.0 cm, deforming the adjacent ribs and displacing the pleura and lung.

The differences between the measurements obtained with each modality reflect the projectional nature of radiography, the limited field of view of ultrasound, and the three-dimensional assessment provided by MRI. The MRI findings assessed against the criteria for well-differentiated liposarcoma are summarized in Table 2.

**Table 2:** MRI findings compared with features that favor well-differentiated liposarcoma. Features favoring well-differentiated liposarcoma are those described by Kransdorf et al. [7]. MRI: magnetic resonance imaging; ADC: apparent diffusion coefficient

Feature	Finding in this patient	Feature favoring liposarcoma
Size	12.0 × 10.2 × 9.1 cm	Greater than 10 cm
Septa	Rare thin septa, less than 2 mm	Thick septa, greater than 2 mm
Nodular or globular non-adipose areas	Absent	Present
Proportion of fat	Greater than 75% of the lesion	Less than 75% of the lesion
Enhancement	No significant enhancement on visual comparison of pre- and post-contrast fat-saturated T1-weighted images	Enhancing septa or nodules
Diffusion	No restricted diffusion; ADC not measured, as there were no non-adipose areas	Not part of these criteria

The imaging findings favored a benign adipocytic tumor, although they could not exclude a lipoblastoma or a well-differentiated liposarcoma. Given the size of the mass, the chest wall deformity, the risk of further skeletal and functional impairment, and the cosmetic impact, surgical resection was performed by the pediatric surgery team through an incision centered over the mass. A large, yellowish, slightly firm tumor was found, covered medially by the serratus anterior muscle and adherent to the chest wall muscles and ribs. It occupied approximately the second to the eighth intercostal spaces and extended into the sixth intercostal space, deforming the upper rib cranially and the lower rib caudally in a U shape and displacing the pleura and lung, without apparent invasion of the pleural cavity. The upper rib of this intercostal space, the sixth rib, showed signs of a consolidated fracture. The tumor was dissected circumferentially from the muscle planes and ribs, and access to the pleural cavity was required to complete the resection. No rib resection was required, as the ribs were deformed but not invaded, and a chest drain was placed. There was no significant blood loss, and there were no intraoperative complications. The postoperative course was uneventful. The chest drain was removed on postoperative day three, and the patient was discharged on postoperative day seven.

Gross examination showed an oval, lobulated, yellow, greasy, and shiny specimen with an irregular multinodular surface and elastic consistency, measuring 13.2 × 10.1 × 10.0 cm and weighing 456 g. The cut surface was homogeneous, soft, and yellow. Histopathological examination showed a lipoma with a focus of ischemic-pattern necrosis and calcification. Margin status was not reported, and immunohistochemical or molecular studies were not performed. Lipoma was therefore considered the most likely diagnosis, but a mature lipoblastoma could not be excluded.

The patient was followed for one year, with no clinical or imaging evidence of recurrence, and was then discharged from follow-up. The timeline of the case is summarized in Table 3.

**Table 3:** Timeline of the case. MRI: magnetic resonance imaging

Time	Event
Age six months	Small palpable left dorsal mass first noticed by the mother
Age about one year	Core needle biopsy at another facility showing mature adipose tissue without atypia; no follow-up considered necessary
Age two years	Referral because of continued growth, delayed by limited access to pediatric surgical care in her region of origin; radiography, ultrasound, and MRI
June 2022	Surgical resection; chest drain removed on postoperative day three; discharge on postoperative day seven
One year after surgery	No clinical or imaging recurrence; discharged from follow-up

## Discussion

Giant lipomas are uncommon in adults and rare in preschool children, with only isolated pediatric cases reported [2,3]. The present tumor met the definition of a giant lipoma by size, measuring 13.2 cm in its largest dimension, although its weight of 456 g was below 1 kg. Although usually asymptomatic, giant lipomas can cause pain from nerve compression, restricted joint motion, and, on the chest wall, skeletal deformity [8]. Resection is indicated in such cases even when the lesion is believed to be benign, and the deep extent of the tumor determines the complexity of the procedure.

Compared with previously reported pediatric cases, this patient combined several unusual features. Giant fatty tumors in children have been described in the thoracoabdominal wall [3] and in the upper extremity [10], whereas the giant lipoblastoma reported in a one-year-old boy was located within the thoracic cavity [11]. In the present case, a chest wall lipoma extended through an intercostal space into the extrapleural compartment, with a transmural growth pattern, U-shaped deformation of the adjacent ribs, and a rib fracture. This pattern shows how a progressively growing adipocytic tumor can reach deep compartments by following the path of least resistance between the ribs, displacing rather than invading the pleura and lung.

The fracture involved the sixth rib, which borders the intercostal space through which the tumor extended and was deformed together with the adjacent rib. On imaging, it was slightly displaced, with signs of ongoing consolidation and without cortical destruction, periosteal reaction, or marrow involvement. At surgery, it showed signs of consolidation; the tumor was dissected from the ribs, no bone invasion was described, and no rib resection was required. There was no history of trauma. Therefore, we consider the fracture most likely secondary to chronic mechanical stress on a rib deformed by the expanding mass rather than a sign of infiltration, although an unrecognized minor trauma cannot be excluded.

In a child of this age, the first differential diagnosis of a fatty mass is lipoblastoma, a benign tumor of embryonal fat that occurs mostly in children younger than five years, predominates in boys, and favors the extremities and trunk [5,6]. Lipoblastoma may reach a large size, may recur after excision, and can mature into a lesion indistinguishable from lipoma [6]. On imaging, it often contains more septa, myxoid areas, and non-adipose tissue than a lipoma, but the overlap is substantial. The distinction rests on histology, which shows lobules of adipocytes at different stages of maturation, lipoblasts, and myxoid stroma [5,6], and molecular testing can support it in equivocal cases, because *PLAG1* rearrangements are found in a large proportion of lipoblastomas [13]. In the present case, the pathology report described a lipoma with a focus of ischemic necrosis and calcification, without a detailed microscopic description, and neither immunohistochemistry nor molecular testing was performed. The homogeneous fatty appearance of the specimen and the absence of myxoid or non-adipose components on MRI favor lipoma, but a mature lipoblastoma cannot be excluded, and the diagnosis of lipoma should be regarded as presumptive. The progressive growth of the mass during the first two years of life is also compatible with lipoblastoma. The biopsy performed one year earlier showed mature adipose tissue, consistent with the final specimen, but a limited sample may not be representative of a large mass.

The second concern is well-differentiated liposarcoma. MRI is the preferred modality for characterizing fatty tumors [1,7]. Features favoring liposarcoma include a lesion size greater than 10 cm, thick septa, nodular or globular non-adipose areas, and a fat component below 75% of the lesion [7]. In our patient, size was the only one of these features present (Table 2). The lesion followed fat signal on all available sequences, showed no restricted diffusion or enhancement, and displaced adjacent structures rather than infiltrating them. However, these criteria were derived mainly from adult series, and overlap between lipoma and well-differentiated liposarcoma has been described [7]. Their applicability to a two-year-old child, in whom liposarcoma is exceptional and lipoblastoma is the main alternative, is therefore limited. The slight heterogeneity seen on ultrasound and T2-weighted images corresponded to rare thin septa, whereas the small focus of necrosis and calcification found in the specimen was not detected on imaging, and no calcification was visible on radiography. Calcification and fat necrosis occur in both lipoma and well-differentiated liposarcoma and do not discriminate between them [7]. MRI can therefore suggest a benign adipocytic lesion and guide management, but it cannot replace histological confirmation.

Other differential diagnoses for a soft-tissue mass at this age and site include hemangioma, juvenile xanthogranuloma, infantile fibrosarcoma, and giant cell fibroblastoma, each with distinctive imaging features [4,9]. Histopathology remains the reference standard.

Surgery was performed about one year after a benign biopsy, when continued growth had already caused skeletal deformity. This interval reflected both the benign biopsy result and a delayed referral, as the patient lived far from the capital, where access to pediatric surgical care is limited, and the demand for pediatric surgery in the public health system is high. A benign biopsy of a growing fatty mass in a young child does not exclude lipoblastoma, and clinical and imaging follow-up after the biopsy may allow earlier recognition of continued growth. Because a mature lipoblastoma could not be excluded and lipoblastoma may recur after excision [6], one year of follow-up is insufficient to confirm a cure. We recommend prolonged clinical and imaging surveillance, including monitoring for chest wall deformity and spinal alignment during growth.

This report has limitations. It describes a single patient, and its findings cannot be generalized. The MRI protocol did not include T1-weighted images without fat saturation, margin status was not reported, histological images were not available, and immunohistochemical and molecular studies were not performed. Follow-up was limited to one year.

## Conclusions

Giant lipoma should be considered in young children with a progressively growing chest wall mass, together with lipoblastoma and liposarcoma. In this patient, MRI showing fat signal on all sequences, no enhancement, no restricted diffusion, and a displacing growth pattern suggested a benign lesion despite its size, deep extrapleural extension, and skeletal changes. These findings can guide surgical planning, but imaging cannot replace histological confirmation, and molecular testing may be required to distinguish lipoma from mature lipoblastoma. Complete resection is indicated for large lesions with skeletal deformity and should be followed by prolonged clinical and imaging surveillance.
